# Modelling the effects of cerebral microvasculature morphology on oxygen transport

**DOI:** 10.1016/j.medengphy.2015.09.004

**Published:** 2016-01

**Authors:** Chang Sub Park, Stephen J. Payne

**Affiliations:** Institute of Biomedical Engineering, Department of Engineering Science, University of Oxford, United Kingdom

**Keywords:** Mathematical modelling, Microcirculation, Oxygen dynamics, Oxygen extraction fraction, Cerebral metabolic rate

## Abstract

The cerebral microvasculature plays a vital role in adequately supplying blood to the brain. Determining the health of the cerebral microvasculature is important during pathological conditions, such as stroke and dementia. Recent studies have shown the complex relationship between cerebral metabolic rate and transit time distribution, the transit times of all the possible pathways available dependent on network topology. In this paper, we extend a recently developed technique to solve for residue function, the amount of tracer left in the vasculature at any time, and transit time distribution in an existing model of the cerebral microvasculature to calculate cerebral metabolism. We present the mathematical theory needed to solve for oxygen concentration followed by results of the simulations. It is found that oxygen extraction fraction, the fraction of oxygen removed from the blood in the capillary network by the tissue, and cerebral metabolic rate are dependent on both mean and heterogeneity of the transit time distribution. For changes in cerebral blood flow, a positive correlation can be observed between mean transit time and oxygen extraction fraction, and a negative correlation between mean transit time and metabolic rate of oxygen. A negative correlation can also be observed between transit time heterogeneity and the metabolic rate of oxygen for a constant cerebral blood flow. A sensitivity analysis on the mean and heterogeneity of the transit time distribution was able to quantify their respective contributions to oxygen extraction fraction and metabolic rate of oxygen. Mean transit time has a greater contribution than the heterogeneity for oxygen extraction fraction. This is found to be opposite for metabolic rate of oxygen. These results provide information on the role of the cerebral microvasculature and its effects on flow and metabolism. They thus open up the possibility of obtaining additional valuable clinical information for diagnosing and treating cerebrovascular diseases.

## Highlights

• Solving for O_2_ transport using artificial models of the cerebral microvasculature.
• O_2_ extraction is dependent on the transit time distribution.
• Metabolic rate is dependent on the transit time distribution.
• Transit time has an effect on the response of metabolic rate to step changes.

## 1 Introduction

Estimates of cerebral blood flow (CBF), mean transit time (MTT), oxygen extraction fraction (OEF) and cerebral metabolic rate of oxygen (CMRO_2_) using imaging modalities have been widely used in clinical studies to diagnose ischaemic lesions, where CBF is the flow of blood per volume of tissue, MTT is the ratio of the cerebral blood volume (CBV) to CBF, with CBV being the volume of blood per volume of tissue, OEF is the fraction of oxygen removed from the blood in the capillary network by the tissue and CMRO_2_ is the product of CBF and OEF. Studies using positron emission tomography have shown regions of brain tissue surviving during ischaemia. Such tissue is characterised by a reduction in CBF and an increase in oxygen extraction to meet the necessary metabolic demand [1], [2]. Detecting such tissues is clinically important, especially in ischaemic stroke, as it helps to determine the ischaemic but still viable tissue, and thus potentially provide useful information during diagnosis and treatment.

Tissue oxygenation is known to occur at both the arteriolar and microvascular level. Since the spatial resolution of imaging modalities does not reach the length scales where tissue oxygenation occurs, models have been developed to determine the effects of the brain microvasculature during neural activity and brain lesions. Generating microvascular structures from real human brain tissue is extremely complex and time consuming as it involves sample preparation, scanning and image processing. Furthermore, the heterogeneity in the morphological properties and the complex arrangements of the vessels (inter-connected and randomly distributed) increases this difficulty. The alternate option is to generate artificial microvascular networks, using specific algorithms [3], [4], matching experimentally obtained morphological properties such as vessel density, length and radius distributions, and connectivity. Su et al. [3] considered the effects of the morphological properties on the transport of blood. Linninger et al. [4] applied this to understand oxygen exchange between blood vessels and brain cells by quantifying oxygen advection in the microcirculation, tissue oxygen perfusion and oxygen consumption. Such artificial networks have been used to develop continuum models of blood flow in capillary network [5] using a multi-scale homogenization method proposed by Shipley and Chapman [6].

Models of oxygen exchange have been developed to examine various physiological effects, for example autoregulation [7], vasomotion (the rhythmic oscillations in blood vessels independent of physiological or neuronal input) [8], [9] or the coupling between CBF and CMRO_2_ [10]. A starting point to many tissue oxygen exchange models in the microvasculature has been the Krogh model [11]. This model represented the entire microvascular network using a single capillary vessel that supplied oxygen to the surrounding cylinder of tissue. Subsequent Krogh-type models have assumed equally spaced parallel vessels to represent the microvasculature and have been mainly considered for skeletal muscles where capillaries tend to run in parallel [12]. The microcirculation in the brain, however, is more complex with the distribution of the capillaries being more random [13]. Thus more realistic and complex vascular geometries than the Krogh-type models are required.

Hsu and Secomb [14] considered a Green’s function approach, where each vessel is treated as a source term, to determine the spatial distribution of tissue oxygenation, assuming that the tissue oxygen consumption is uniform and using the Hill equation to account for the nonlinear oxygen dissociation. The model was subsequently extended to relax several of the assumptions considered. A review of the method and its extensions is available in Secomb et al. [15]. The results showed that tissue oxygen level is highly heterogeneous and thus can lead to tissue hypoxia at lower oxygen consumption rate than predicted using Krogh-type models with equivalent average capillary density.

Models of oxygen delivery have attempted to provide a better understanding of the coupling between CBF and CMRO_2_. Buxton and Frank [10] considered a model of oxygen transport from the capillary to the tissue to predict a high coupling ratio due to physiological limitations of oxygen delivery. A single vessel model was considered assuming steady state oxygen concentration, constant oxygen permeability and ratio of plasma to blood oxygen concentration, and negligible tissue oxygen concentration. Hyder et al. [16] introduced dynamic changes in oxygen permeability to provide a better fit to *in vivo* data of changes in CBF and CMRO_2_. Hayashi et al. [17] considered varying oxygen diffusivity and nonlinear binding of haemoglobin to oxygen. Zheng et al. [18] included dynamic changes in oxygen transport as well as relaxing the assumption of negligible tissue oxygen concentration.

More recently, there has been an interest in the heterogeneity of the flow patterns and the effects of the transit time distribution. Jespersen and Østergaard [19] considered a non-physiological collection of parallel equal length capillary vessels with an underlying transit time distribution (a gamma distribution) to determine the maximum available oxygen concentration and its effects with changes in transit time distribution using a Hill’s equation approximation for the nonlinear dissociation of oxygen and with the assumption that tissue oxygen concentration is negligible. The results show that, even for a simple network, a complex nonlinear behaviour in OEF and CMRO_2_ with both individual and combinational changes in mean and variance of the transit time distribution can be observed. The assumption of a negligible tissue oxygen concentration was relaxed by Angleys et al. [20] by assuming that the rate of oxygen metabolism in the tissue is governed by the Michaelis–Menten kinetics. Several different transit time distributions were considered to show a similar complex nonlinear behaviour in OEF and CMRO_2_ previously observed by Jespersen and Østergaard [19]. However, given the inter-connectedness of the capillary network and studies suggesting flow distribution and thus transit time distribution being significantly dependent on network topology [21] and network density [22], any *in vivo* comparison will require models with morphologically accurate microvascular networks replicating physiology.

Studies with artificial microvascular networks attempting to replicate physiological morphology have suggested that the topology of the cerebral microvasculature appears to play an important role in the adequate supply of oxygen to the cerebral tissue, which will have an important role during ischaemia. In this paper, a dynamic oxygen transport model was developed and applied to artificially generated capillary networks with matching morphological properties, previously developed by Su et al. [3] to quantify the relationship between MTT, mean absolute deviation transit time (MADTT), OEF and CMRO_2_ with varying morphological properties. Generating many networks based on a specific algorithm to run the simulations creates a heterogeneous sample of networks. This approach has enabled us to extract further information about the microvasculature, which may have potential clinical value for diagnosis and treatment.

## 2 Theory

Artificial capillary networks are generated here to solve for oxygen transport. A minimum spanning tree algorithm is used to randomly generate physiologically accurate capillary networks [3] based on the matching of histological data from experiments [13], [23]; these include matching vessel density, length and radius distributions, inlet and outlet densities, and node connectivity. Su et al. [3] solved for the flow assuming Poiseuille flow with individual vessel viscosity obtained from an experimental relationship [24] by applying a pressure gradient and taking the net flow at the nodes to be zero. Once the flow distribution is determined, oxygen concentration in the capillary network can be obtained by solving the mass transport equation. The approach considered here is an extension to that proposed by Park and Payne [22] to solve for the residue function in microvascular networks assuming that the concentration is driven solely by convection, with the extension proposed here being the addition of a tissue diffusion term with a rate proportional to the difference in oxygen concentration between the plasma and the tissue. Assuming that oxygen concentration in the tissue, *C_t_*, is constant and that the oxygen dissociation is linear so that the fraction of oxygen concentration in plasma to blood, *r*, is constant along the capillary vessels, the one-dimensional mass transport for a single vessel can be expressed as:

(1) $$\frac{\partial C(x,t)}{\partial t}+U\frac{\partial C(x,t)}{\partial x}=-\lambda \left[C\left(x,t\right)-C_{T}\right]$$

where *C* is the oxygen concentration, *x* is the axial coordinate, *t* is time, *U* is the velocity, *λ* is the product of the surface permeability and *r*, and $C_{T}=C_{t}/r$. Considering $C^{\prime }\left(x,t\right)=C\left(x,t\right)-C_{T}$ in Eq. (1) and taking the Laplace transform, with $C^{\prime }\left(x,t=0\right)=0$ due to initial steady-state conditions, *C*′ can be expressed in the *s*-domain as:

(2) $$C^{\prime }\left(x,s\right)=C^{\prime }\left(0,s\right)\exp\left[-\frac{(s+\lambda )x}{U}\right]$$

where *s* is the complex frequency variable. The oxygen concentration at the outlet in the time-domain, for a given inlet concentration, can be obtained by taking the inverse Laplace transform of Eq. (2). Considering a unit step function, *u*(*t*), for simplicity, with a magnitude of *C*_0_ at the vessel inlet, such that $C\left(0,s\right)=C_{0}/s,$ the outlet oxygen concentration can be expressed in time-domain as:

(3) $$C\left(L,t\right)=\left(C_{0}-C_{T}\right)u\left(t-T\right)e^{-\lambda T}+C_{T}u\left(t\right)$$

where *L* is the length of the vessel and T=L/U is the transit time. Since the change in concentration is convection driven, the outlet oxygen concentration is a simple step function with a delay *T*, and a tissue oxygen concentration term that determines the baseline oxygen concentration in the vessel. The magnitude of the outlet oxygen concentration then depends on the amount of oxygen diffused into the tissue, this being the difference in oxygen concentration between the vessel inlet and tissue.

For a capillary network, it is necessary to consider the distribution of oxygen concentration at the nodes where the vessel junctions occur. The concentration at each node is taken to be the sum of all supplying vessels weighted with the flow rates:

(4) $$C_{j}^{\prime }\left(s\right)=\frac{\sum_{i}C_{ij}^{\prime }\left(L_{ij},s\right)Q_{ij}}{\sum_{i}Q_{ij}}$$

where *Q* represents flow rate. The subscripts represent the nodes being considered; *j* represents node *j* whilst *ij* represents from node *i* to node *j*. Note that nodes *i* and *j* are only considered if these are connected as the parameters will be equal to zero when the nodes are not connected. From Eq. (4), the outlet concentration of the network is dependent on the individual outlet node concentrations and the ratio of the outlet node flow to the net flow, *Q_net_*, and can be expressed in the *s*-domain as:

(5) $$C_{v}\left(s\right)=\frac{C_{T}}{s}+\sum_{a,v}\frac{Q_{nv}}{Q_{net}}\left[\left(C_{a}\left(s\right)-\frac{C_{T}}{s}\right)\prod_{i=a,j}^{j=v}\frac{Q_{ij}}{\sum_{k}Q_{kj}}e^{-\left(s+\lambda_{ij}\right)T_{ij}}\right]$$

where subscripts *a, v, n* and *k* represent the network nodes for the inlet, outlet, the nodes connected to the outlet nodes and node *k* respectively. Note that in addition to the network topology, *λ* and *C_T_* are the other parameters required to determine the outlet oxygen concentration.

Taking the inverse Laplace transform of Eq. (5) with a given inlet concentration function, it is possible to obtain the outlet concentration in the time-domain. OEF can then be obtained from:

(6) $$\text{OEF}\left(t\right)=\frac{C_{a}\left(t\right)-C_{v}\left(t\right)}{C_{a}\left(t\right)}$$

and CMRO_2_ can be obtained from:

(7) $${\text{CMRO}}_{2}\left(t\right)=\text{CBF}\cdot C_{a}\left(t\right)\cdot \text{OEF}\left(t\right)$$

## 3 Results and discussion

Three different sized network cubes are considered here, with 100 different networks with equivalent conditions randomly generated for each cube size to allow for the heterogeneity. The mean value of the morphological properties obtained from each cube size was matched to morphological properties obtained from experimental results [13], [23]. Table 1 shows the morphological properties of the three different cube sizes. The number of vessels and total length of each cube size are obtained per litre of tissue for comparison purposes. Note that there is no significant difference in the morphological properties between the three cube sizes. The standard deviation decreases with increasing cube size as the heterogeneity of the networks within the same cube size decreases. An inlet flow and an outlet pressure were set as the boundary conditions. All the inlet and outlet nodes were set to the same flow and pressure respectively. The sum of the individual flow at the inlet nodes is set to equal the CBF value to be considered whilst the outlet pressure is set to be the same at all the outlet nodes.

**Fig. 1:**
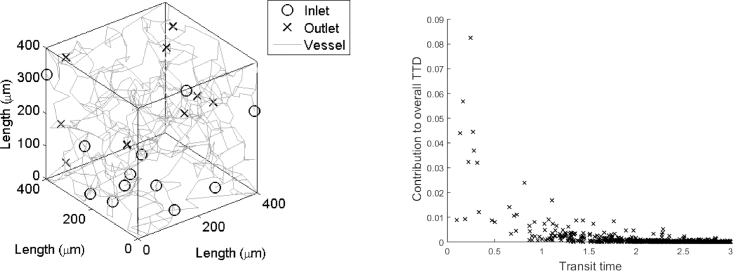
A sample capillary network and its respective transit time distribution (TTD) shown up to transit time values of 3 s.

Fig. 1 shows a sample capillary network and its respective transit time distribution, obtained according to the method proposed by Park and Payne [22]. The transit time distribution for the sample capillary network, which shows the contribution of the transit time distribution for all the possible pathways from the inlets to the outlets, is shown up to transit time values of 3 s. Each marker in the transit time distribution represents a pathway with a set transit time and its overall contribution determined by the product of the ratio of the nodal inflow contribution from the vessel to the net inflow at each node along the pathway. The transit time distribution is positively skewed showing similarities to a gamma distribution, but with a very long tail suggesting that it is very heterogeneous. A step change in the concentration at the inlet is considered:

(8) $$C_{a}\left(t\right)=\alpha \left[1+\beta u\left(t-\tau \right)\right]$$

where *α* is the magnitude of the concentration at the inlet, *β* is the percentage change in the magnitude and *τ* is the time at which the step change is considered. The concentration at the outlet can be obtained by taking the Laplace transform of Eq. (8), substituting this term into Eq. (5) and taking the inverse Laplace transform. Substituting this term and Eq. (8) into Eq. (6), OEF can be expressed as:

(9) $$\text{OEF}\left(t\right)=1-\frac{1}{C_{a}\left(t\right)}\left[C_{T}+\sum_{a,v}Y_{av}\frac{Q_{nv}}{Q_{net}}\prod_{i=a,j}^{j=v}\frac{Q_{ij}}{\sum_{k}Q_{kj}}e^{-\lambda_{ij}T_{ij}}\right]$$

where $Y_{av}=\left(\alpha -C_{T}\right)u\left(t-\sum_{i=a,j}^{j=v}T_{ij}\right)+\left(\beta -C_{T}\right)u\left(t-\sum_{i=a,j}^{j=v}T_{ij}-\tau \right)$. CMRO_2_ can then be obtained by substituting the value of CBF, Eqs. (8) and (9) into Eq. (7).

Table 2 shows the cube size and the initial conditions driving the flow and concentration between the inlets and outlets. CBF was set to be similar to a normal average value of cerebral blood flow, measured in ml of blood per 100 ml of tissue per minute, in adults [25], 55 ml/100 ml/min, unless stated otherwise. Baseline oxygen concentration, measured in *µ*mol of oxygen per ml of blood, at the vessel inlet and tissue was set to 9 *µ*mol/ml and 0.03 *µ*mol/ml respectively [26]. *r* was set to its mean value of 0.01 and λ=1 was chosen for all vessels so that OEF would be around the baseline OEF value of 0.4. A constant inlet concentration was first considered (β=0) followed by a step decrease in the magnitude of the inlet concentration (β=−0.2).

Table 3 shows the steady state mean and standard deviation of CBV, MTT, MADTT, OEF and CMRO_2_ of the three cube sizes considered for a constant inlet concentration. There is no significant difference between the mean values in CBV, MTT, MADTT, OEF and CMRO_2_ for the three cube sizes as expected since these are determined by the morphological, blood and tissue properties, which were matched for all cube sizes. CBV and CMRO_2_ are measured in ml of blood per ml of tissue and *µ*mol of oxygen per ml of tissue per minute respectively. Note that the standard deviation in each of the values obtained is largest for the smaller cube. This is probably due to a greater degree of heterogeneity in the morphological properties of the created networks at the smaller length scale. MTT and MADTT can be calculated from the transit time distribution. MADTT is considered here instead of the more typical standard deviation as the transit time distribution is heavily positively skewed with a very long tail making the standard deviation prone to outliers. For each network, changes in inlet flow will change both MTT and MADTT in the same way such that an increase in CBF will cause a drop in both MTT and MADTT. These two parameters cannot be uncoupled as they are obtained from the same transit time distribution, which for a passive network like the one considered here, depends only on the inlet conditions and the morphological properties.

**Fig. 2:**
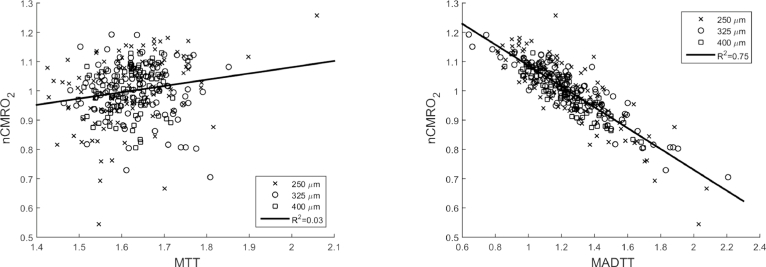
Plot of MTT and MADTT against mean normalised CMRO_2_ with CBF = 55ml/100ml/min for the three different cube edge lengths; 250, 325 and 400 *µ*m.

Fig. 2 shows the variation of mean normalised CMRO_2_ (nCMRO_2_) with MTT and MADTT. A linear regression is considered here to quantify the correlation between the parameters due to the simplicity in the analysis and by no means establishes that the two parameters have a linear relationship. Since both *C_a_* and CBF are kept constant, the plot of OEF will only differ with the plot of CMRO_2_ in magnitude and thus it is not shown here. No trend can be observed between MTT and nCMRO_2_ ($R^{2}=0.03$) due to the small difference in MTT between the different networks. There is however, a negative correlation between MADTT and nCMRO_2_ ($R^{2}=0.75$), and thus with MADTT and OEF. The latter observation is in agreement with the result obtained by Jespersen and Østergaard [19] with their simplistic network of parallel equal length capillary vessels with a transit time following a gamma distribution where, for a constant MTT, OEF increased with decreasing variance of the transit time. This is due to the overall magnitude of the concentration at the outlet depending on the sum of all the individual pathway transit times as well as MTT. In the case of MADTT and CMRO_2_, both Jespersen and Østergaard [19], and Angleys et al. [20] showed that for a transit time following a gamma distribution, this would increase up to a certain value in the variance of the transit time and decrease once passed this value. Angleys et al. [20] showed that this was the case for other distributions too, but not for all distributions considered. Given a CBF value, each artificial network presented here produces a specific transit time distribution. Hence the relationship between the parameters in passive artificial networks can be determined without the need to assume for a transit time distribution.

**Fig. 3:**
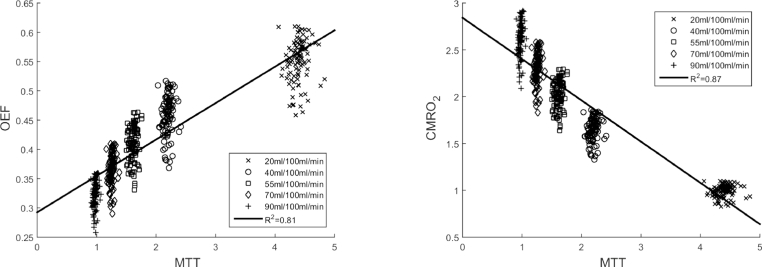
Plot of MTT against OEF and CMRO_2_ with varying CBF for cube edge length of 400 *µ*m.

A change in CBF was next considered to determine the effects of changes in MTT. Fig. 3 shows the variation of MTT with OEF and CMRO_2_ respectively for the 100 artificial networks generated with a cube size of 400 *µ*m. Note that MTT has a positive correlation with MADTT for changes in CBF as both MTT and MADTT are obtained from the same transit time distribution, determined by network topology. Hence Fig. 3 implicitly considers changes in MADTT too. The spread in OEF values increases with increasing MTT suggesting that the heterogeneity of the network is more significant at higher MTT. Although the parameters on both plots show signs of a nonlinear relationship, a linear regression is once again considered to quantify the correlation between the parameters. A positive correlation can be observed between MTT and OEF ($R^{2}=0.81$) and a negative correlation can be observed between MTT and CMRO_2_ ($R^{2}=0.87$). As MTT increases as a result of a decrease in CBF, OEF will increase as there is more time for oxygen to be extracted to maintain CMRO_2_, even without any feedback. However, OEF increase is constrained by the amount of oxygen available and thus CMRO_2_ will eventually drop as CBF decreases.

Variations in transit time distribution have shown to vary OEF and CMRO_2_. From the linear regression gradients obtained in Figs. 2 and 3, it is possible to obtain the sensitivity of the ratio of the change in OEF to baseline OEF to the ratio of the change in MTT to baseline MTT and the ratio of the change in MADTT to baseline MADTT:

(10) $$\frac{\Delta OEF}{\bar{OEF}}=S_{1}\frac{\Delta MTT}{\bar{MTT}}+S_{2}\frac{\Delta MADTT}{\bar{MADTT}}$$

where the overline represents the baseline value of the parameter, determined in Table 3, and *S*_1_ = 0.42 and *S*_2_ = −0.18 are the sensitivity due to changes in MTT and MADTT. The positive and negative signs determine the direction it changes. As |*S*_1_| > |*S*_2_|, changes in MTT have a greater contribution to changes in OEF than changes in MADTT. The opposite signs determine that OEF increases and decreases with increasing MTT and MADTT respectively. This relationship is in agreement with the nonlinear behaviour observed in Fig. 3, where increasing negative changes in CBF from baseline causes decreasing positive changes in OEF from baseline.

The above approach can also be considered for CMRO_2_:

(11) $$\frac{\Delta CMRO_{2}}{\bar{CMRO_{2}}}=S_{1}\frac{\Delta MTT}{\bar{MTT}}+S_{2}\frac{\Delta MADTT}{\bar{MADTT}}$$

where *S*_1_ = 0.08 and *S*_2_ = −0.43. Since |*S*_1_| < |*S*_2_|, changes in MADTT have a greater contribution to changes in CMRO_2_ than changes in MTT. The opposite signs determine that CMRO_2_ increases and decreases with increasing MTT and MADTT respectively. This relationship is in agreement with the nonlinear behaviour observed in Fig. 3, where increasing positive changes in CBF from baseline causes decreasing negative changes in CMRO_2_ from baseline.

Angleys et al. [20] showed that for a transit time following a gamma distribution, CMRO_2_ could increase or decrease with the heterogeneity of the transit time distribution for a constant MTT. This was the general trend for the transit time distributions considered but not for all those considered. Changes in both *C_T_* and *C_a_* were next considered. From the OEF expression in Eq. (9), it was found that changes in $\left(C_{a}-C_{T}\right)/C_{a}$ keeping CBF constant led to a linear change in OEF and thus CMRO_2_.

The coupling between CBF and CMRO_2_ during brain activation, still a subject of debate, has been characterised by calculating the ratio of fractional CBF changes to fractional CMRO_2_ changes. Several experimental studies have been reported this value to be in the range of 2–4 [27], [28], although there have been reports of larger values [26]. For the range of CBF values considered here (20–90 ml/100 ml/min), taking CBF = 55 ml/100 ml/min and its respective CMRO_2_ as reference, the ratio of fractional CBF changes to fractional CMRO_2_ changes was found to be between 1.2 and 2.2; lower than the reported values. It was also found, using the OEF expression in Eq. (9), that this ratio varied with $C_{a}-C_{T},$ hence the initial and boundary concentration conditions have a significant contribution to the solution. Note that this is a passive network, suggesting that the difference in the reported values with those found here could be due to the active feedback.

**Fig. 4:**
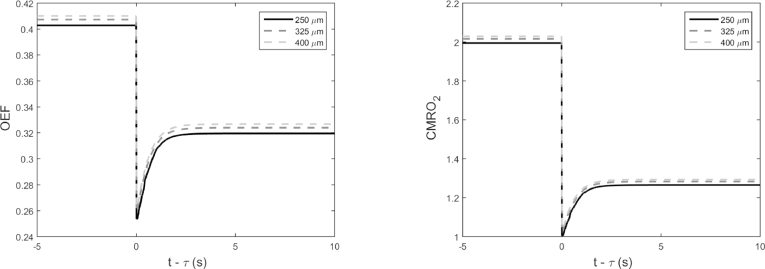
Mean OEF and CMRO2 with dynamic changes in the concentration at the inlet for the three different cube edge lengths; 250, 325 and 400 *µ*m.

In order to observe the response of the network for a dynamic change, an artificial step decrease function in the inlet concentration is considered at t=τ. Fig. 4 shows the effects of the step change in mean OEF and CMRO_2_. For each cube size, a drop of 20% oxygen concentration at the inlet causes a sharp decrease in both mean OEF and mean CMRO_2_, gradually returning back to 80% and 64% of their original steady state value respectively as expected. The time for this recovery to occur for all cubes is less than 3s. An exponential recovery curve fitted to each plot shows the time constant to be approximately 0.6 s. Thus cube size does not have an effect on the recovery. Once more, using the OEF expression in Eq. (9), the exponential recovery time constant was found to be dependent on $\left(C_{a}-C_{T}\right)/C_{a}$.

A limitation of the theoretical method considered here is that the equations need to be linear. Furthermore, several assumptions were considered in the model, these being constant oxygen permeability along each of the capillary vessel, constant ratio of plasma to blood oxygen concentration and constant tissue oxygen concentration. The assumption of a constant oxygen permeability across each of the capillary vessel was initially kept constant due to the significant variability between the studies [26], leading to simpler mathematics and analysis of the results. The assumption that the tissue oxygen concentration across the cube is constant was considered here since the spatial resolution of imaging modalities cannot reach microvascular levels. Estimates of the different imaging markers were obtained by taking the average signal of the voxel considered.

Although the oxygen dissociation curve is nonlinear, with the ratio of plasma to blood oxygen concentration varying between 0.008 and 0.015 along the capillary vessel length with a mean value of 0.01, a linear approximation has been previously used [10], [18] and is considered here. The nonlinear oxygen binding to haemoglobin has been approximated by the Hill equation [17], [29], [30]. Zheng et al. [18] compared the linear oxygen dissociation with the Hill equation approximation and showed that the linear approximation was appropriate over the physiological baseline flow range and flow changes. This was explained by the fact that oxygen haemoglobin concentration is much greater than the oxygen plasma concentration. Since the model considered here is considering a time-averaged solution, the mean value was considered to be a good approximation.

A recent experimental work performed on mouse cerebral cortex showed that the parenchymal arterioles are responsible for 50% of oxygen exchange at baseline conditions and the rest takes place within the first few capillary branches [31]. The latter finding can be observed using the capillary network model considered here. Eq. 5 shows that the oxygen concentration at the nodes are dependent on $\prod_{i,j}\exp\left(-\lambda_{i,j}T_{i,j}\right)=\exp\left(-\sum_{i,j}\lambda_{i,j}T_{i,j}\right)$. The transit times will increase as the branching order increases, thus leading to an exponential decay in the oxygen concentration. Taking a constant λ=1 across the network and assuming that vessels only split during the capillary branches, the concentration half-life can be estimated to *T*_1/2_ ≃ 0.43. The mean transit time of the capillaries, $T_{mean}≃O\left(1\right),$ thus most of the oxygen exchange will occur within the first few capillary branches.

The aim of this work is for clinical applications in pathological conditions such as ischaemic stroke. This will require comparing the results obtained with clinical results. Although the preliminary results show that cube size does not affect the solution, any direct comparison with clinical data will still require matching it to image voxel sizes as the cubes considered here consider vessels linking from neighbouring cubes in an implicit manner. The model here thus implicitly assumes that the tissue region receives its oxygen supply only from the vessels within it. Scaling up the cubes generated here will require a different approach. El-Bouri and Payne [5] applied the multi-scale homogenization method proposed by Shipley and Chapman [6] to develop continuum models of blood flow in a capillary network model of the human cortex. This work was extended to include the mass transport effects [32]. Implementing effects such as oedema [33], swelling of cells, observed in stroke can provide useful information during diagnosis and treatment. This will be the subject of future work.

## 4 Conclusion

An extension to a recently developed mathematical technique to solve for the residue function in a capillary network with matching physiological topology has been developed here to solve for OEF and CMRO_2_. The results show that the volume of the tissue considered does not affect the solution as long as the morphological properties are matched. However, great care needs to be taken when directly comparing biomarkers obtained here with clinical data as the artificial cube considered here does not have vessels linked to neighbouring cubes. Sensitivity analysis of the perfusion parameters MTT and MADTT were performed to quantify their respective contribution to changes in OEF and CMRO_2_. For OEF, the contribution of changes in MTT (S = 0.42) was found to be greater than changes in MADTT (S = −0.18). For CMRO_2_, the contribution of changes in MTT (S = 0.08) was found to be less than changes in MADTT (S = −0.43). Positive changes in both MTT and MADTT contributed to positive and negative changes respectively to both OEF and CMRO_2_. For the dynamic case, a step change in the concentration at the inlet leads to a sharp change in both OEF and CMRO_2_ followed by a gradual recovery. Once again, this recovery is dependent on the properties of the transit time distribution. Note that a passive network was considered here, with no feedback. Such passive models could be used to separate the different contributions of the passive response and the active feedback caused by a change in flow or neural activity. Furthermore, these networks can be used to develop effects such as oedema observed in stroke and assemble these detailed components to construct the higher-level processes.

## Conflicts of Interest

None declared.

## Ethical Approval

Not required.

## Figures and Tables

**Table 1:** Network morphological properties.

Parameter	Edge length (*µ*m)		
	250	325	400
Number of vessels (/l tissue)	7761 ± 729	7752 ± 551	7769 ± 547
Inlet:Outlet	3:2	7:5	12:9
Total length (mm/l tissue)	466.8 ± 27.8	465.4 ± 19.5	463.6 ± 15.9
Node connectivity, M (%):			
M = 3	95.2 ± 1.58	95.6 ± 0.94	95.8 ± 0.72
M = 4	4.8 ± 1.58	4.4 ± 0.94	4.2 ± 0.72
Diametre (*µ*m) a		6.235 ± 1.285	

Mean ± standard deviation.

a The same diametre distribution was considered for all three cube sizes.

**Table 2:** Initial conditions for the networks considered.

Parameter	Value	Reference
CBF (ml/100ml/min)	55	[25]
*α* (*µ*mol/ml)	9	[26]
*β*	0, −0.2	
*λ* (/s)	1	
*C_T_* (*µ*mol/ml)	0.03	[26]
*r*	0.01	[18]

**Table 3:** Parameter values for the initial conditions considered.

Parameter	Edge length (*µ*m)		
	250	325	400
CBV (ml/ml)	0.0148 ± 0.0009	0.0150 ± 0.0007	0.0148 ± 0.0005
MTT (s)	1.61 ± 0.10	1.64 ± 0.08	1.62 ± 0.06
MADTT (s)	1.26 ± 0.26	1.26 ± 0.26	1.21 ± 0.18
OEF	0.40 ± 0.05	0.41 ± 0.04	0.41 ± 0.03
CMRO_2_ (*µ*mol/ml/min)	2.00 ± 0.24	2.02 ± 0.19	2.03 ± 0.15

Mean ± standard deviation.
